# Screening and Identification of Prognostic Tumor-Infiltrating Immune Cells and Genes of Endometrioid Endometrial Adenocarcinoma: Based on The Cancer Genome Atlas Database and Bioinformatics

**DOI:** 10.3389/fonc.2020.554214

**Published:** 2020-12-01

**Authors:** Bingnan Chen, Di Wang, Jiapo Li, Yue Hou, Chong Qiao

**Affiliations:** ^1^ Department of Obstetrics and Gynaecology, Shengjing Hospital of China Medical University, Shenyang, China; ^2^ Key Laboratory of Obstetrics and Gynecology of Higher Education of Liaoning Province, Shenyang, China; ^3^ Department of Internal Medicine, Shengjing Hospital of China Medical University, Shenyang, China

**Keywords:** endometrioid endometrial adenocarcinoma, tumor-infiltrating immune cells, prognosis, The Cancer Genome Atlas, bioinformatics

## Abstract

**Background:**

Endometrioid endometrial adenocarcinoma (EEA) is one of the most common tumors in the female reproductive system. With the further understanding of immune regulation mechanism in tumor microenvironment, immunotherapy is emerging in tumor treatment. However, there are few systematic studies on EEA immune infiltration.

**Methods:**

In this study, prognostic tumor-infiltrating immune cells (TIICs) and related genes of EEA were comprehensively analyzed for the first time through the bioinformatics method with CIBERSORT algorithm as the core. Gene expression profile data were downloaded from the TCGA database, and the abundance ratio of TIICs was obtained. Kaplan–Meier analysis and Cox regression analysis were used to identify prognostic TIICs. EEA samples were grouped according to the risk score in Cox regression model. Differential analysis and functional enrichment analyses were performed on high- and low-risk groups to find survival-related hub genes, which were verified by Tumor Immune Estimation Resource (TIMER).

**Result:**

Four TIICs including memory CD4+ T cells, regulatory T cells, natural killer cells and dendritic cells were identified. And two hub gene modules were found, in which six hub genes including *APOL1, CCL17, RBP4, KRT15, KRT71*, and *KRT79* were significantly related to overall survival and were closely correlated with some certain TIICs in the validation of TIMER.

**Conclusion:**

In this study, four prognostic TIICs and six hub genes were found to be closely related to EEA. These findings provided new potential targets for EEA immunotherapy.

## Introduction

Endometrial carcinoma is one of the most common tumors in the female reproductive system. Ranking fifth most common malignancies, endometrial carcinoma is diagnosed in about 300,000 individuals worldwide every year (1). A study initiated by the Centers for Disease Control in 2018 showed that the morbidity and mortality of endometrial carcinoma have increased in 26 countries and regions over the past decade (2). Endometrioid endometrial adenocarcinoma (EEA) is the most common type of endometrial carcinoma, accounting for about 70–80% (3). The survival rate of EEA patients can reach 85%, but it is less than 50% for the patients in advanced-stage with low benefits in present therapeutic strategies (4, 5). The development of new treatment methods to further improve the survival rate of EEA patients is an urgent problem to be solved in the clinic.

In recent years, it is shown that immune cells and inflammatory factors have played an important role in the growth, invasion, and proliferation of malignant tumors (6). Tumor-infiltrating immune cells (TIICs), as a part of tumor microenvironment, are abundantly present in the matrix of EEA, which affect the development of EEA (7). The study of TIICs in EEA is of great significance to the immunotherapy of EEA (8). However, most studies on the TIICs in EEA mainly focus on a single sample or a single immune cell (9, 10), which cannot provide comprehensive assessments. Therefore, to systematically evaluate the potential immunotherapy targets of EEA, the data of patients with EEA were downloaded from the TCGA database, and the prognostic TIICs and related hub genes of EEA were identified through bioinformatics method in this study.

## Materials and Methods

### Data Preparation and Download

All RNA-seq gene expression profiles and clinical data of patients with EEA were downloaded from TCGA (https://portal.gdc.cancer.gov/). Gene expression data in FPKM format were then normalized by R software 3.6.2 (R Foundation for Statistical Computing, Vienna, Austria). In total, there were 406 samples with EEA in the study.

### Abundance Ratio Matrix and Correlation of Tumor-Infiltrating Immune Cells

CIBERSORT is an approach for analyzing immune cell composition of complex tissues from their gene expression profiles (11). We download CIBERSORTR source code and LM22 gene signature from CIBERSORT web portal (http://cibersort.stanford.edu/) and run it locally. The abundance ratio matrix of 22 TIICs was obtained from gene expression profiles of EEA. The matrix was visualized by barplot and pheatmap packages in R. In total, 252 EEA samples were selected with p-value <0.05 as the cut-off value.

### Identifying the Relationship Between Tumor-Infiltrating Immune Cells and Clinical Characters

Totally 251 samples were selected with both clinical data and matrix of TIICs. The relationship between the abundance ratio of TIICs and clinical characters including tumor grade and clinical stage was analyzed by Pearson correlation analysis with corrplot package in R.

### Identifying Survival-Related Tumor-Infiltrating Immune Cells and Grouping

Survival analysis was conducted, and survival curves were plotted using Kaplan–Meier analysis by the log-rank test which was performed by survival package in R. The LASSO is a regression analysis method that performs both variable selection and regularization (12). It was performed through glmnet package and survival package in R with the number of lambda = 1000 in order to evaluate the correlation between TIICs and overall survival of EEA patients. Then the overlapped TIICs in LASSO were selected to perform multivariate Cox regression analysis. Immune cells with the p-value <0.05 were identified as independent prognostic factors. Risk score was calculated for each sample by prognostic TIICs expression levels of Cox regression model. Samples were separated into high- and low-risk groups determined by the median score as a cut-off. Also the nomogram was constructed in R for evaluating prognosis by prognostic TIICs. C-index of Cox regression model was calculated, and calibration curve was drawn with rms package in R.

### Identifying Differentially Expressed Genes and Enrichment Analysis

DEGs between the high- and low-risk groups were selected by edgeR package in R with p-value <0.05 and fold change >1.5 as a filter. Then Gene Ontology (GO) (13) and Kyoto Encyclopedia of Genes and Genomes (KEGG) (14) enrichment analyses and visualization of DEGs were performed by clusterprofiler, enrichplot, and org.Hs.eg.db package in R with p-value <0.05 as the cut-off value. Gene set enrichment analysis (GSEA) was performed using GSEA software v3.0 (15). Immunologic signatures’ gene set (C7 gene sets) was selected as a reference in Molecular signatures database (MSigDB) for GSEA (http://software.broadinstitute.org/gsea/msigdb).

### Protein–Protein Interactions Network Construction and Hub Module Analysis

To construct protein interactions’ network and find hub gene modules, STRING database (v11.0) (16) (https://string-db.org/) was used, and the combined-score was set to ≥0.4. Then molecular complex detection (MCODE) plugin in Cytoscape software (17) was used to select clusters that included 10 or more nodes. ClueGo App was used to perform GO and KEGG enrichment analyses for top two clusters with the most nodes selected by MCODE. The clusters were identified as hub gene modules. Pearson correlation analysis between genes in hub modules and clinical characters was carried out. Six hub genes in modules were screened out through Kaplan–Meier survival analysis.

### Validation of the Six Hub Genes

Six hub genes were verified at the mRNA level by real-time PCR. Totally 16 endometrial EEA samples and eight normal endometrium samples were obtained. All samples were obtained with the consent of the patients. Primer pairs used for each hub gene were shown in **Supplementary Table 1**. Total RNA of each sample was extracted using RNAiso Plus (TaKaRa, Beijing, China) and then reverse transcribed using HiScript^®^ III RT SuperMix for qPCR (Vazyme, Nanjing, China). ChamQTM Universal SYBR^®^ qPCR Master Mix (Vazyme, Nanjing, China) was used to quantitatively detect mRNA level of six hub genes in real-time PCR with the β-actin gene as an endogenous control for mRNA normalization. ΔΔCt method was used for analysis of the real-time data. F-test and T-test were used to analyze differences in mRNA levels between groups.

### Validation of the Immune Correlation

Pearson correlation analysis was used to analyze the correlation between hub genes and TIICs which was obtained by the CIBERSORT. The Tumor Immune Estimation Resource 2.0 (TIMER 2.0) (http://timer.cistrome.org/) (18) provides more robust estimation of immune infiltration levels for The Cancer Genome Atlas (TCGA) using six state-of-the-art algorithms (19). It can be used to perform Spearman’s correlation analysis between multiple TIICs and specific gene, which is used to validate the immunologic correlation in this study.

## Results

### The Tumor-Infiltrating Immune Cell Infiltration Landscape in Endometrioid Endometrial Adenocarcinoma

**Figure 1:**
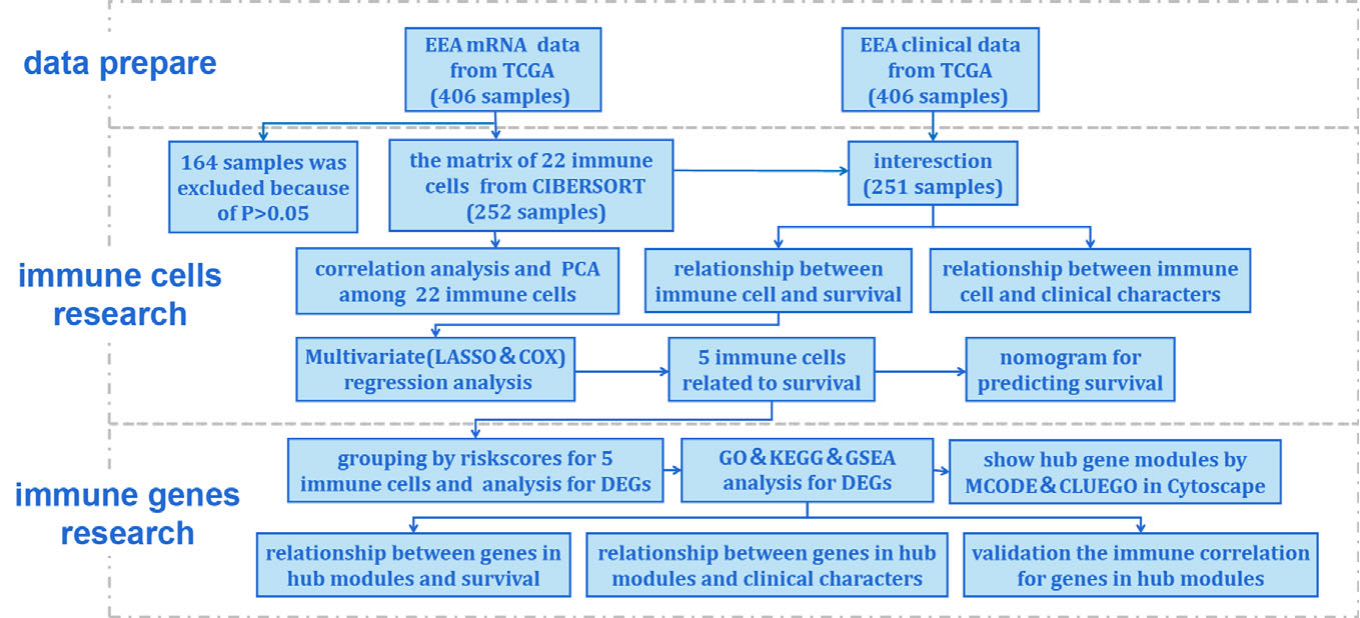
Flow chart of the data processing procedures. UCEC, uterine corpus endometrial carcinoma. TCGA, the Cancer Genome Atlas (https://portal.gdc.cancer.gov/). CIBERSORT, a method for analysis immune cells composition of complex tissues from their gene expression profiles (http://cibersort.stanford.edu/); PCA, principal component analysis; LASSO, least absolute shrinkage and selector operation regression analysis; COX, Cox proportional hazards regression analysis; DEGs, differentially expressed genes; GO, Gene Ontology; KEGG, Kyoto Encyclopedia of Genes and Genomes; GSEA, Gene Set Enrichment Analysis; MCODE, molecular complex detection plugin in Cytoscape software; CLUEGO, an app for enrichment analysis in Cytoscape software.

**Figure 2:**
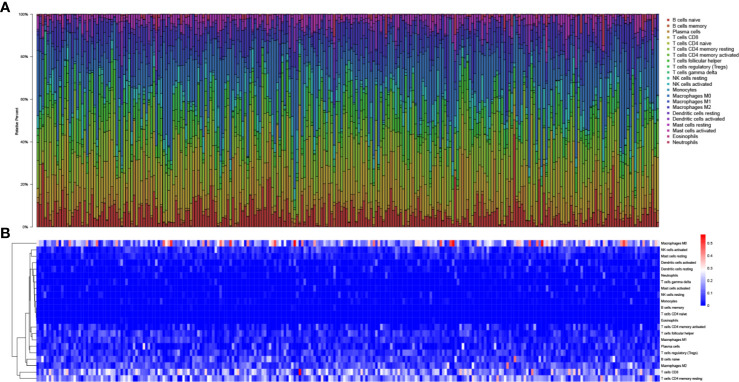
The landscape of tumor-infiltrating immune cells. **(A)** Bar plot of the fraction of tumor-infiltrating immune cells; **(B)** Heat map of the fraction of tumor-infiltrating immune cells.

In total, 425 EEA samples in TCGA were enrolled in the study. The subsequent analyses were shown in **Figure 1**. 252 EEA samples were selected with p-value <0.05 as the filter in CIBERSORT algorithm (**Supplementary Table 2**). The abundance ratio matrix of 22 TIICs in the 252 samples was shown in **Figures 2A, B**. Naive CD4+ T cell was excluded in the following analyses because the abundance was 0 in each sample.

### Impacts of Tumor-Infiltrating Immune Cell Abundance Ratio on Clinical Characteristics

**Figure 3:**
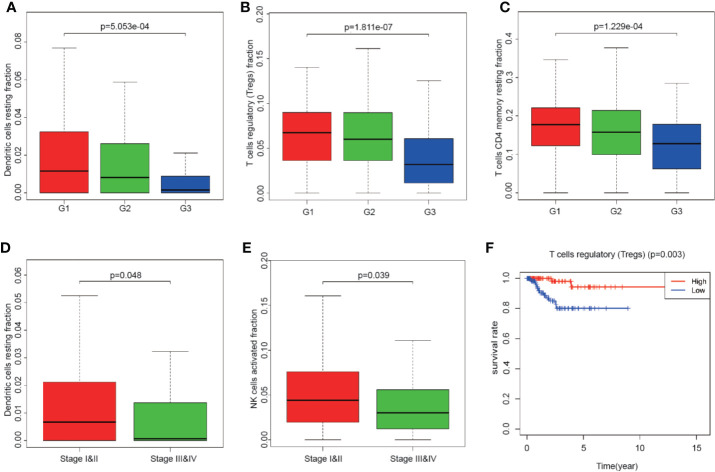
The relationship between clinical characters and overall survival with the fraction of tumor-infiltrating immune cells. **(A**–**C)** The relationship of the fraction of tumor-infiltrating immune cells and EEA clinical grades; **(D, E)** The relationship of the fraction of tumor-infiltrating immune cells and EEA clinical stages; **(F)** Kaplan–Meier survival curve for the abundance ratios of tumor-infiltrating immune cells.

Totally 251 samples with both clinical data and TIICs abundance ratio matrix were selected. The clinical characteristics including age, BMI, vital status *etc*. are listed in **Table 1**. Correlation between TIICs abundance ratio and clinical characters was performed by Wilcoxon signed-rank test with p-value <0.05 as the cut-off value. There was a significant negative correlation between the clinical grade of EEA and fraction of resting dendritic cells (p < 0.001), Tregs (p < 0.001) and resting memory CD4+ T cells (p < 0.001) (**Figures 3A–C**), while the fraction of resting dendritic cells (p = 0.048) and activated natural killer cells (NK cells) (p = 0.039) showed a significant negative correlation with the clinical stage of EEA (**Figures 3D, E**).

**Table 1:** Clinical characteristics of 251 samples.

Characteristics	Alive (n = 236)	Dead (n = 15)	P value
Age	62.03 ± 10.97	64.4 ± 14.86	0.387
BMI	35.07 ± 9.54	33.84 ± 7.52	0.793
Stage			0.005
I	169	6	
II	20	0	
III	38	7	
IV	9	2	
Grade			0.021
G1	64	0	
G2	67	4	
G3	105	11	

### Identification of Survival-Related Tumor-Infiltrating Immune Cells

**Figure 4:**
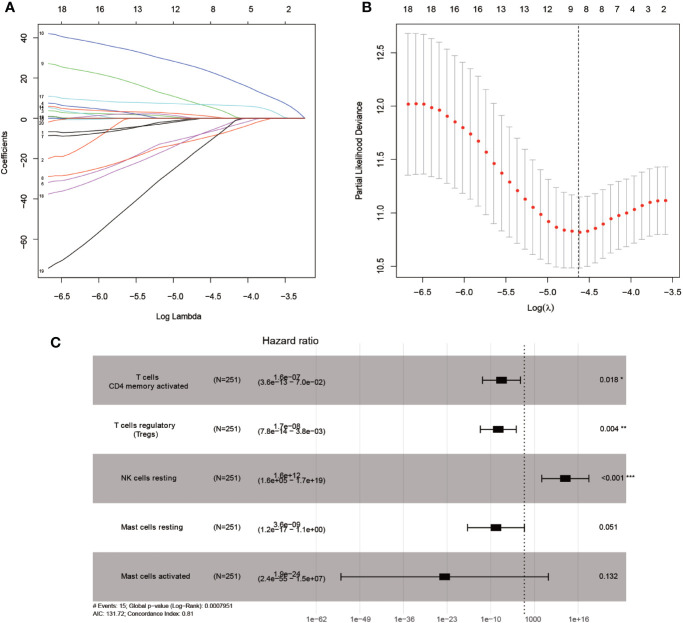
Identifying prognostic tumor-infiltrating immune cells by multivariate analysis. **(A, B)** LASSO regression coefficient profiles of prognostic tumor-infiltrating immune cells; **(C)** The forest map of multivariate the Cox regression analysis.

**Figure 5:**
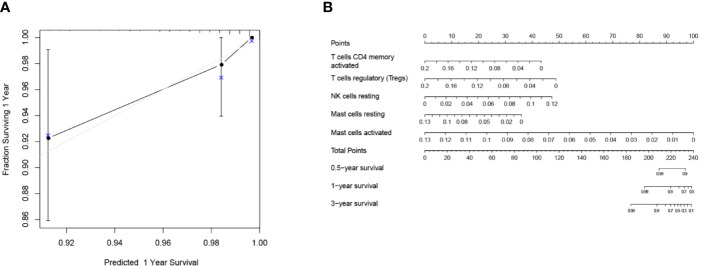
Validation and application of the Cox regression model. **(A)** Calibration curve of the Cox regression model; **(B)** The nomogram for predicting 3-year survival rates of EEA.

Mainly two methods were used to depict the relationship between overall survival and TIICs in EEA, Kaplan–Meier survival analysis and Cox regression analysis. All TIICs were separately included in Kaplan–Meier survival analysis. And the result showed that the fraction of Tregs was significantly related to survival (p < 0.001) (**Figure 3F**). Meanwhile, all TIICs were preliminarily screened through LASSO algorithm prior to Cox regression analysis, and nine TIICs were shown to be possibly related to survival, including naive B cells, activated memory CD4+ T cells, Tregs, gamma delta T cells, resting NK cells, macrophage M1, activated dendritic cells, resting mast cells, and activated mast cells (**Figures 4A, B**). After that, Cox regression analysis was performed using these nine TIICs, five of which were selected into the Cox regression model. Activated memory CD4+ T cells (p = 0.018), Tregs (p = 0.004), and resting NK cells (p < 0.001) were identified as independent prognostic factors in the Cox regression model (**Figure 4C**). C-index of Cox regression model was 0.814, and calibration curve was drawn, which shows the strong discrimination and calibration power of the model (**Figure 5A**). A nomogram based on the Cox regression analysis was developed for prognostic prediction of EEA (**Figure 5B**).

### Identification of Differentially Expressed Genes and Functional Enrichment

**Figure 6:**
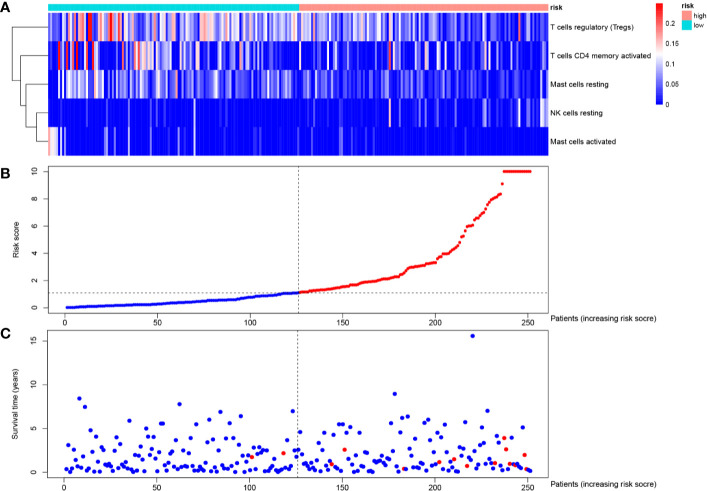
Risk curve of the Cox regression model. Distribution of **(A)** risk score, **(B)** survival statuses and **(C)** expression profile of prognostic tumor-infiltrating immune cells of each patient.

**Figure 7:**
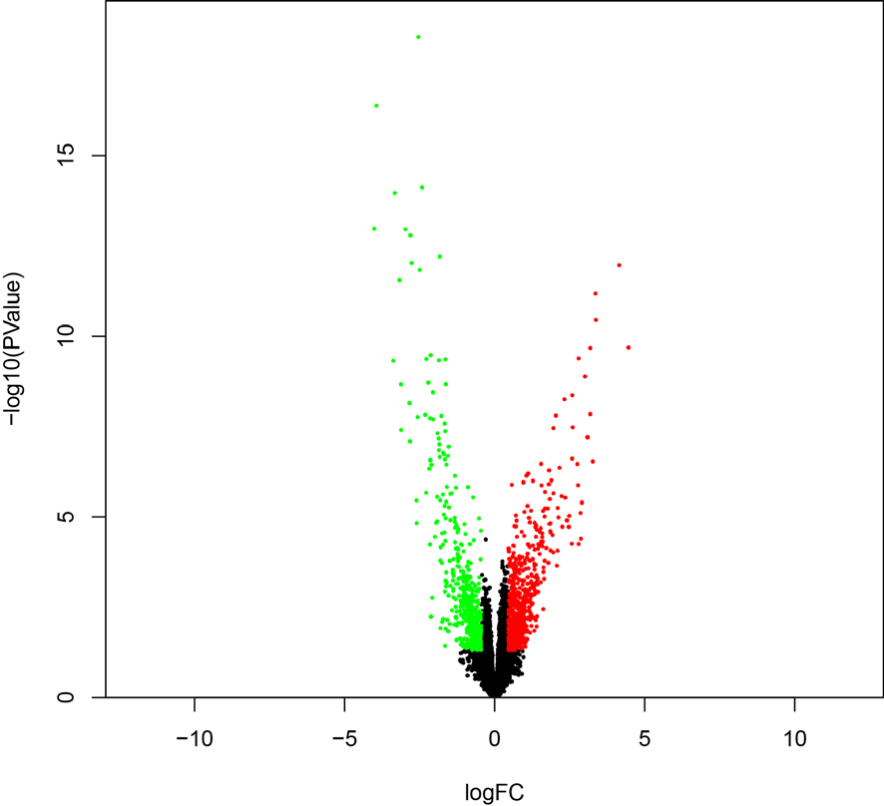
The volcano plot of DEGs between high- and low-risk groups.

**Figure 8:**
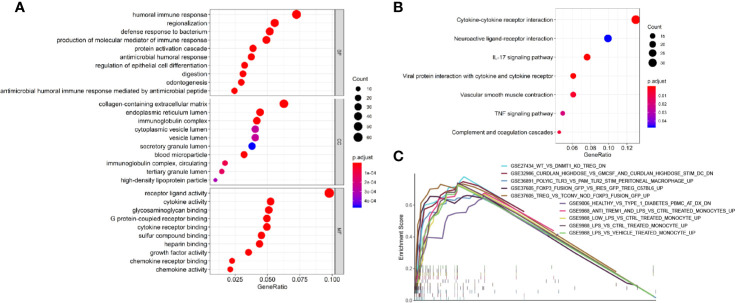
Enrichment analyses of immune-related DEGs. **(A)** The top 10 of biological process GO terms, cellular component GO terms, molecular function GO terms; **(B)** Significantly enriched KEGG pathways of immune-related DEGs; **(C)** The 10 top-ranked gene sets of GSEA.

To identify survival-related DEGs in EEA, 251 samples were divided into low- and high-risk groups with the median risk score generated in Cox regression analysis as the cut-off value (**Figures 6A–C**) (**Supplementary Table 3**). The R package “edgeR” was then used to identify immune-related DEGs between low- and high-risk groups and totally 1,276 DEGs were found (p < 0.05, fold change > 1.5, detailed information shown in **Supplementary Table 4**). Volcano plot of DEGs was shown in **Figure 7**. Multiple functions and pathways related to immune response and cytokines were enriched in Gene ontology (GO) and Kyoto Encyclopedia of Genes and Genomes (KEGG) enrichment analyses (**Figures 8A, B**) (**Supplementary Tables 5**, **6**), such as the humoral immune response, immunoglobulin complex, and cytokine-cytokine receptor interaction. To further confirm that DEGs were related to immune function, Gene set enrichment analysis (GSEA) with the immunologic signatures gene sets (C7 gene sets) was performed to further explore the immune function of DEGs, in which 244 gene sets were selected and 81 gene sets were significantly enriched (p < 0.05, shown in **Supplementary Table 7**). The top 10 gene sets with normalized enrichment scores were shown in **Figure 8C**.

### Protein–Protein Interactions Network Construction and Hub Module Analysis

**Figure 9:**
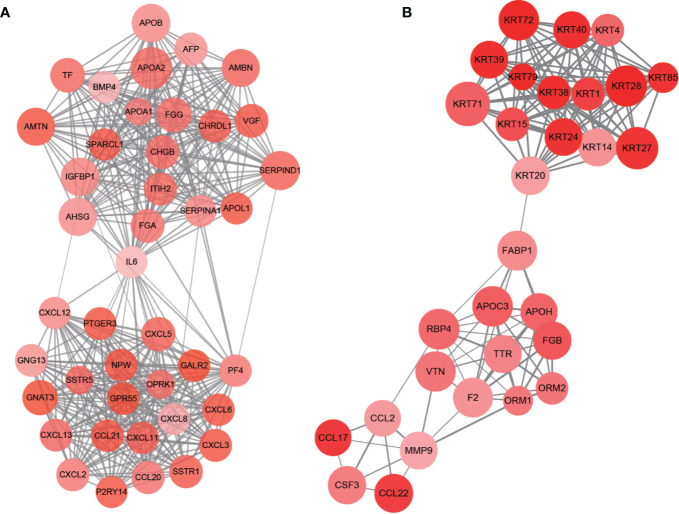
PPI network of hub modules. Module 1 **(A)** and module 2 **(B)** are the top two modules (>10 nodes) in the PPI network. The size of nodes associates with the log(FC) value, the color reflects the clustering coefficient, and edge width reflects the combined score.

**Figure 10:**
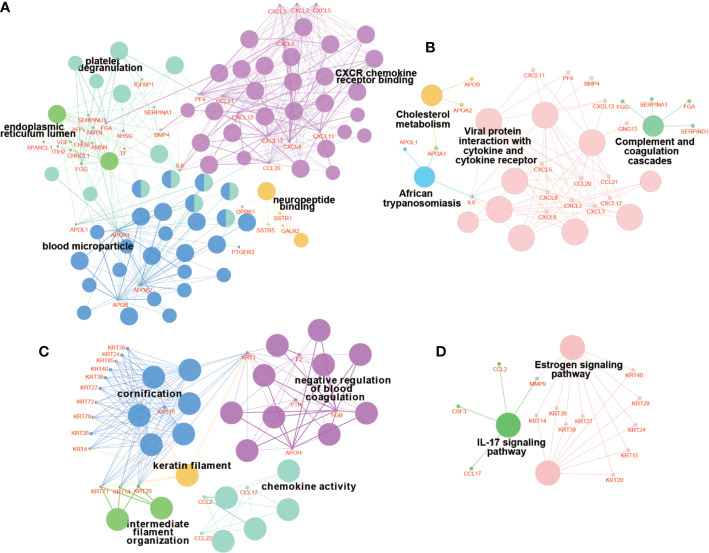
Enrichment analyses of genes in hub modules. The results of GO enrichment analysis of genes in module 1 **(A)** and module 2 **(C)**. The results of KEGG enrichment analysis of genes in module 1 **(B)** and module 2 **(D)**. The bigger nodes indicate pathways and terms, while the smaller nodes indicate genes involved. And the color reflects the functional group of pathways and terms.

**Figure 11:**
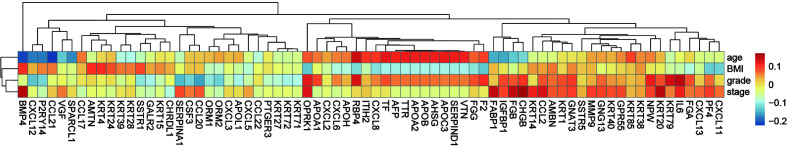
The correlation between genes in hub modules and clinical characters.

PPI network of DEGs was constructed by STRING, in which 31 modules were obtained through cluster analysis of MCODE plugin in Cytoscape software. The top two hub modules were selected and performed GO and KEGG enrichment analyses by ClueGo (**Figures 9**, **10**). The detailed result of enrichment analyses was shown in **Supplementary Table 8**. The results demonstrated that the first hub module was mainly enriched in platelet degranulation (35.71%), CXCR chemokine receptor binding (33.33%), and blood microparticle (28.57%), and the second hub module was mainly enriched in cornification (50%) and Estrogen signaling pathway (33.33%). The results of correlation analysis between clinical characters and the genes in two hub modules were shown in **Figure 11**. The Kaplan–Meier survival analysis showed that six hub genes including *APOL1* (p = 0.031), *CCL17* (p = 0.043), *RBP4* (p = 0.037), *KRT15* (p = 0.030), *KRT71* (p = 0.018), *KRT79* (p = 0.022) were significantly associated with overall survival, which could be potential immunotherapy targets.

### Validation of the Six Hub Genes

**Figure 12:**
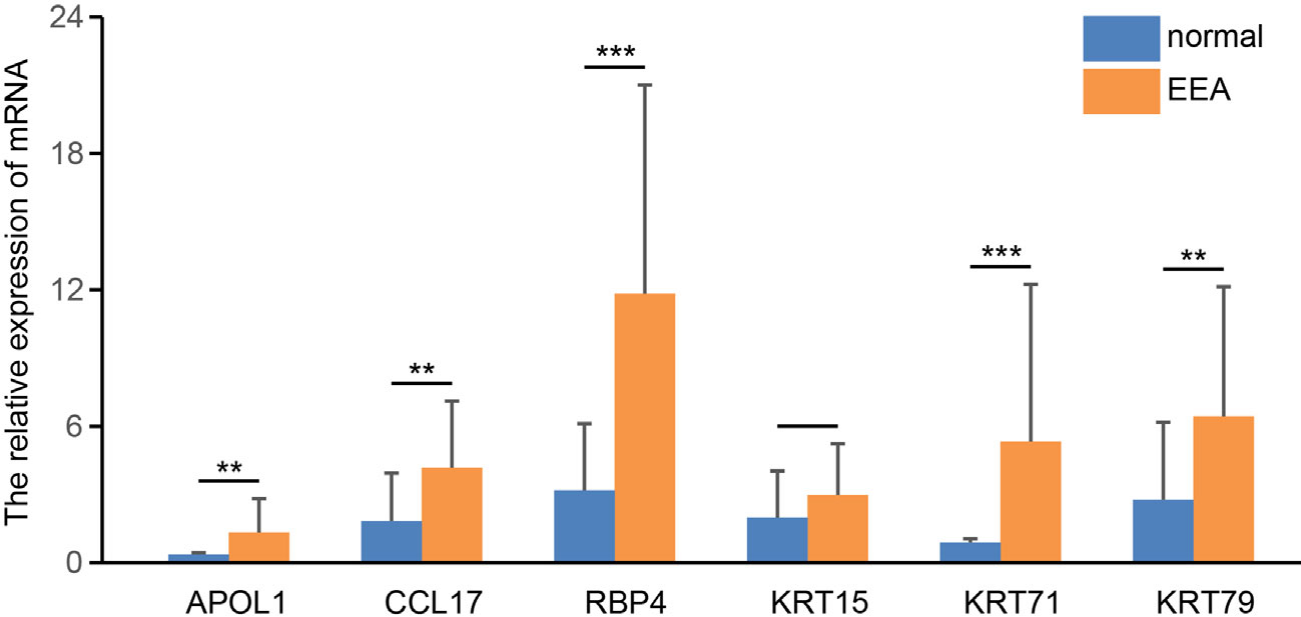
The relative expression level of 6 hub genes in EEA and normal samples. ** and *** indicate p < 0.01, p < 0.001, respectively.

We detected the mRNA expression levels of six hub genes in EEA samples and normal endometrial samples by real-time PCR. The results showed that the expression levels of four genes in EEA samples, including APOL1, CCL17, RBP4, and KRT71, were significantly higher than those in normal endometrial samples (p < 0.05, see in **Figure 12**). This is consistent with their expression trends in high- and low-risk groups, that is, expression level in high-risk groups is higher than that in low-risk groups. The expression level of KRT15 did not show a significant difference between EEA samples and normal endometrial samples. The expression level of KRT79 in EEA samples is higher, but according to the above results, the expression level of KRT79 in the low-risk group is higher, which is inconsistent with its expression trend between EEA and normal samples.

### Validation of the Immune Correlation

**Figure 13:**
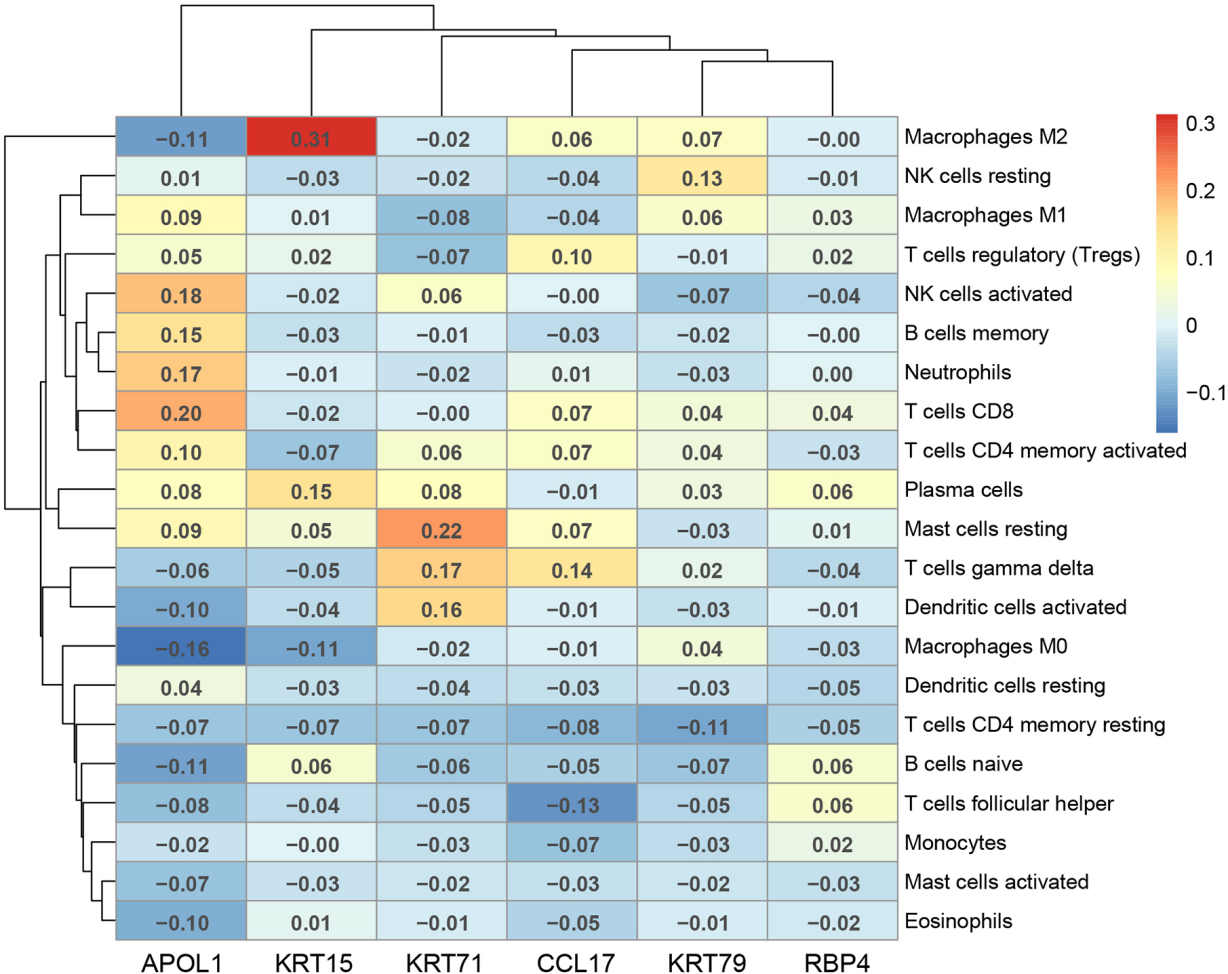
The Pearson correlation analysis between the hub genes and tumor-infiltrating immune cells.

**Figure 14:**
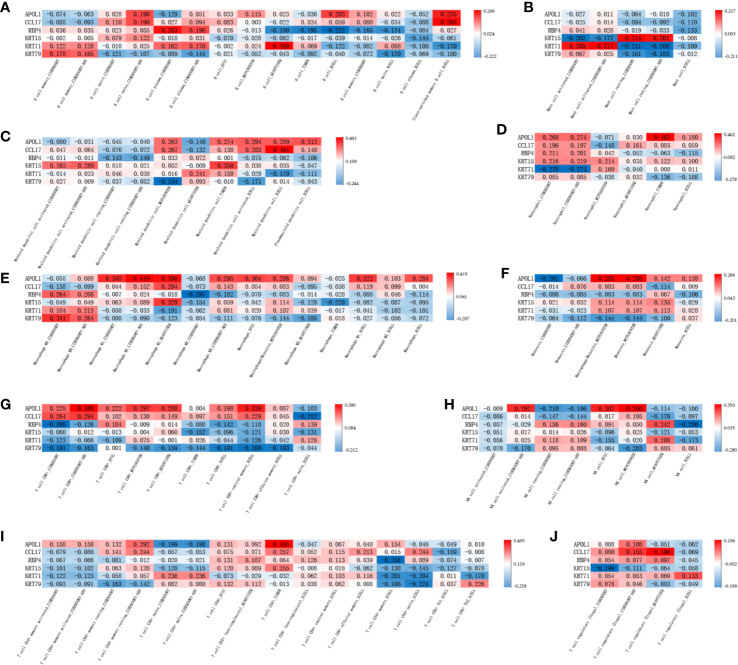
The validation in TIMER 2.0 between the hub genes and tumor-infiltrating immune cells. **(A–J)** shows the correlation between each tumor-infiltrating immune cell and 6 hub genes in different algorithms.

The correlation of the above six hub genes with 21 types of TIICs in EEA was further analyzed, which was shown in **Figure 13**. Then TIMER 2.0 was used to validate the correlation between six hub genes and TIICs using six state-of-the-art algorithms. The results showed that six hub genes were all correlated with some certain TIICs (**Figure 14**). Among them, nine kinds of TIICs including CD4+ T cell (r = 0.495), neutrophil (r = 0.462), macrophage M1 (r = 0.419), CD8+ T cell (r = 0.380) *etc*. are all related to APOL1, which is closely related to TIICs. The detailed correlation coefficient (r > 0.2) of hub genes was shown in **Supplementary Table 9**.

## Discussion

The biological behavior of malignant tumors is closely related to the surrounding immune microenvironment. Immune cells’ infiltration and inflammatory factors have played an important role in the development and invasion of tumors (6, 20, 21). At present, immune studies have been at the forefront of tumor research, but studies on the cellular level are still mainly carried out through immunohistochemistry. Due to technical limitations, these studies were always limited by the small sample size and few cell types, and it was difficult to complete the quantitative detection of proteins. The CIBERSORT algorithm based on bioinformatics is a good way to overcome these shortcomings. It can obtain the landscape of the immune response at the level of 22 immune cells through high-throughput gene expression profiling data. Thus, it is possible to study more immune subtypes precisely in the scope of big data.

In this study, the composition of TIICs in the EEA microenvironment was obtained through the CIBERSORT algorithm for the first time, and a series of analyses were performed to find prognosis-related TIICs and hub genes, providing new targets for EEA immunotherapy.

TIICs were analyzed at multiple levels in this study, and a total of four TIICs were found to be significantly associated with clinical characters and overall survival, including memory CD4+ T cells, Tregs, NK cells, and dendritic cells. And the first three of TIICs were identified as independent prognosis-related TIICs through Cox regression analysis.

It is well known that the maturation of dendritic cells can be achieved through the pathogen-associated molecular patterns (PAMPs) and damage-associated molecular patterns (DAMPs). The mature dendritic cells combine with major histocompatibility complex (MHC) class I and II molecules for antigen presentation. This process acts as the first signal to activate CD8 + and CD4 + T cells. The co-stimulatory interaction between CD28 on T cells and CD80 or CD86 on antigen presenting cells serves as a second signal which is also necessary for T cell activation. In addition, T cells also need other cytokine signals (the third signal) to achieve the survival of effector cells and memory cells, and initiate immune response (22, 23). However, the occurrence and development of many tumors are accompanied by the expression of immunosuppressive cytokines and the loss of these three signals, resulting in the failure of immune responses (24–26). We believe that the lack of the above three signals plays a very important role in the immune escape process of EEA. This study found that memory CD4+ T cells, activated NK cells, and dendritic cells all decreased with the increase of EEA grade and stage, that is, the deletion of the three signals caused by the decrease of these three TIICs was indeed related to the occurrence and development of EEA. In the Cox regression analysis model, memory CD4+ T cells can be used as an independent prognostic protective factor, and NK cells resting can be used as an independent prognostic risk factor. This also further illustrated this point.

Tregs constitute the basic balance of the adaptive immune response, which can adjust its function and homeostasis characteristic according to different tissue environments and immune conditions (27). Numerous Tregs in malignant tumor tissue usually indicate poor clinical outcomes (28). However, the function of Tregs in endometrial cancer is still controversial (29). Giatromanolaki et al. found that Treg cells were not associated with survival in patients with endometrial cancer and lack prognostic significance (30). Besides, Kubler et al. found that tumor-associated macrophages (TAMs) and Tregs acted synergistically in endometrial cancer; however, only TAMs increased in advanced FIGO stages and high tumor grade, while the level of Tregs did not change significantly (31). In this study, the results showed that the infiltrated level of Tregs was positively correlated with survival rate of EEA and negatively correlated with clinical grade. In other words, high levels of Tregs indicated a good prognosis. The reason may be that EEA is an estrogen-dependent tumor and in high-grade EEA, high levels of estrogen affect the infiltration level of Tregs, which was also mentioned in Kubler’s study (31).

In addition, 251 samples were divided into low- and high-risk groups in this study to identify survival-related genes and functions. Totally six hub genes that were significantly related to overall survival were obtained in low- and high-risk groups. Among them, four genes, including *APOL1, CCL17, RBP4*, and *KRT71* were selected in real-time PCR experiments between tumor samples and normal samples, which were found to be consistent with the expression trend in low- and high-risk groups. This indicates that the above four genes may not only participate in the development process of EEA from low risk to high risk, but also participate in the occurrence process of EEA from normal to cancer. Besides, these genes have also been reported to be involved in other various malignant tumors. *APOL1*, as an apolipoprotein, plays a protective role in hepatocellular carcinoma and renal cells carcinoma by mediating tumor cells death (32, 33). In addition, *APOL1* also participates in the innate immune response as an immune effector (34). *CCL17* is a lymphocyte chemokine that plays an important role in the transport and activation of T cells. Tumor-associated neutrophils released cytokine *CCL17*, which can recruit Treg cells into the tumor microenvironment to affect antitumor immunity (35, 36). The overexpression of *APOL1* and *CCL17* in EEA may also improve the prognosis of patients by enhancing these specific functions. *RBP4* is an adipokine that has been reported to drive the migration of ovarian cancer cells (37). KRT71 is a member of the Keratin family, which may be related to hair follicle morphogenesis, but its relationship with cancer has not been reported yet (38).

However, as we showed in the results, the expression trend of KRT79 in real-time PCR experiments is not consistent with that in low- and high-risk groups. Studies have shown that KRT79 is involved in cell migration and tube generation (39), and we speculate that it may affect the tumor angiogenesis. However, we have not verified it in low- and high-risk groups. In future experiments, we would like to use transcriptome sequencing and deconvolution analysis through the CIBERSORT algorithm to verify its expression.

Similarly, the study of Chen (40) also discussed prognostic-related genes in the immune microenvironment of endometrial cancer. Different from Chen’s study, our study only included samples with EEA, while all types of endometrial cancers including sarcoma were included in Chen’s study, which is why the final results of our study differ from Chen’s study. In addition, Chen’s study uses the Estimate algorithm to study immune infiltration, while our study uses the CIBERSORT algorithm. The two algorithms are different in deconvolution principle, which may also cause differences in results. Compared with estimation algorithm, CIBERSORT algorithm and the combination of Lasso and Cox regression analysis are superior because CIBERSORT algorithm can accurately analyze the types and distribution of 22 kinds of immune cells in samples and not just to get a score of the immunocyte invasion of the sample, which increases the accuracy of the results.

However, this study also has some limitations. This study used publicly available data sets from TCGA, which were not considered for immune infiltrating studies when they were collected. Therefore, patients with immune system diseases may be included in this study, which may cause selection bias in the results. In addition, all the patients in this study were selected retrospectively, and the exact location of samples was not known. Therefore, it was difficult to ignore the influence of the number and type of immune cells caused by sampling at the edge of the tumor or within the tumor.

## Conclusion

In this study, we obtain the landscape of the immune environment in EEA at the level of 22 immune cells through high-throughput gene expression profiling data in TCGA. Totally four TIICs and six hub genes related to the prognosis of EEA were screened out through the bioinformatics method with CIBERSORT algorithm as the core, providing new potential targets for EEA immunotherapy.

## Data Availability Statement

Publicly available datasets were analyzed in this study. This data can be found here: https://portal.gdc.cancer.gov/.

## Ethics Statement

The studies involving human participants were reviewed and approved by the Medical Ethics Committee of China Medical University. The patients/participants provided their written informed consent to participate in this study.

## Author Contributions

BC and DW participated in the study design and manuscript drafting. YH took part in the acquisition of data. JL and BC partook in the analysis and interpretation of data. CQ contributed to the study supervision. All authors contributed to the article and approved the submitted version.

## Funding

This study was supported by the National Key R&D Program of China (2016YFC1000404), the National Natural Science Foundation of China (General Program; 81370735), the National Natural Science Foundation of China (General Program; 81771610), and the Outstanding Scientific Fund of Shengjing Hospital (201706), the Distinguished Professor of Liaoning Province (2017).

## Conflict of Interest

We declare that the research was conducted in the absence of any commercial or financial relationships that could be construed as a potential conflict of interest.

## Abbreviations

EEA, Endometrioid endometrial adenocarcinoma; TIICs, tumor-infiltrating immune cells; TCGA, The Cancer Genome Atlas; FPKM, Fragments Per Kilobase of transcript per Million mapped reads; Tregs, regulatory T cells; LASSO, Least Absolute Shrinkage and Selector Operation regression analysis; DEGs, differentially expressed genes; GO, Gene Ontology; KEGG, Kyoto Encyclopedia of Genes and Genomes; GSEA, Gene Set Enrichment Analysis; PPI, Protein–protein interactions; MCODE, molecular complex detection plugin in Cytoscape software; UCEC, Uterine Corpus Endometrial Carcinoma.
